# Supplementary material for: Stable characteristics of intrapopulation heterogeneity in virus-specific Th1 cells during chronic viral challenge infection
Source: Front Immunol. 2025 Dec 19;16:1716422. doi: 10.3389/fimmu.2025.1716422 (PMC12757370; doi:10.3389/fimmu.2025.1716422)
Supplement: Supplementary file 1 [file Table1.pdf]

	symbol	High_S1	High_S2	High_S3	Low_S1	Low_S2	Low_S3	Mock_S1	Mock_S2
ENSMUSG00000001444	Tbx21	35949,3	36798	31585,8	16397,8	19334	17530,8	28949	25348,2
ENSMUSG00000055170	Ifng	17384,8	11637,5	12644	9595,4	6535,1	7911,9	15594,2	11631,6
ENSMUSG00000050232	Cxcr3	38748	43725,5	35560,7	34561,2	39603,4	34371,5	38054	36867,3
ENSMUSG00000079018	Ly6c1	303,8	331,1	246,3	158,6	217,2	225,4	252,5	222,9
ENSMUSG00000022584	Ly6c2	70750,1	77631,1	58948,4	27235,7	27361,7	24298,5	54812,8	51654,3
ENSMUSG00000026068	Il18rap	35771,6	34606,8	32482,4	15885,5	17588,2	13348,6	26490,2	25973,9
ENSMUSG00000026070	Il18r1	13974,3	14154	11268,5	7741,2	7708,8	7096,7	10669,5	10660,9
ENSMUSG00000020009	Ifngr1	21046,5	23175	20124,6	9447,6	9929,2	10768,1	16600,6	15737,4
ENSMUSG00000038151	Prdm1	2494,9	2667,1	2670,4	783,8	936,5	947,7	1747,4	1725
ENSMUSG00000020644	Id2	78966,5	69786,1	58038,2	28258,6	26076,7	27273,9	48474,5	50605,4
ENSMUSG00000062939	Stat4	9890,7	11163,9	8456,9	8164,9	8833,8	7823,1	8419,5	8737,6
ENSMUSG00000000791	Il12rb1	3011	3181	3770,4	2354,1	3054,1	3078,2	2936,7	2973,3
ENSMUSG00000018341	Il12rb2	2428,4	2739,5	2591,5	1249,6	1648,3	1521	2074,2	2069
ENSMUSG00000026770	Il2ra	465,1	669,1	682,4	193,5	263,8	322,5	341	456,4
ENSMUSG00000048163	Selplg	79976,2	94097	79163,5	42548,8	50649,1	49210,7	67623,7	65647,4
ENSMUSG00000037944	Ccr7	1365,2	1423,2	1137,1	10429,4	10331,7	8746,9	4946	4949
ENSMUSG00000015437	Gzmb	12617,7	10035	10677,5	2416,8	2528,6	3348,1	7718,6	6629
ENSMUSG00000048521	Cxcr6	67449,1	61385,9	54938,5	20347,3	20353,1	22622,8	46038,1	41866,7
ENSMUSG00000049103	Ccr2	34452,1	28807	27907,8	10185,7	9352,9	10685,8	21716,5	21309,9
ENSMUSG00000079227	Ccr5	2925,5	2526,8	2081,4	596,6	549,8	566	1568	1403,6
ENSMUSG00000022508	Bcl6	453,1	367,9	618,2	1103,6	1174,8	1284,1	791,7	692,2
ENSMUSG00000027718	Il21	44,9	26,4	61,3	311,7	257,4	440,1	135,7	121,1
ENSMUSG00000047880	Cxcr5	420,3	533,4	491,6	4426	4573,2	4194,5	1968	2035,7
ENSMUSG00000000782	Tcf7	46120,5	44362,4	43673,9	89416	91564	91147,8	65318,2	64930,6
ENSMUSG00000041272	Tox	2478,5	2736,1	2010,3	5681,8	5149,4	4988,4	3503	3142,5
ENSMUSG00000074607	Tox2	30,2	52,9	73	1144,8	1425,9	1684,7	539,2	520,7
ENSMUSG00000055435	Maf	3442,4	3722,4	3281,7	2664	2566,8	2410,3	2857,6	3061,1
ENSMUSG00000007872	Id3	709,4	425,4	529,6	6601,8	4040,3	4869,9	2832,8	1971,5
ENSMUSG00000026009	Icos	16140,4	18059,3	12411,4	21931	20919,8	19705	18202,9	17734,5
ENSMUSG00000027947	Il6ra	987,3	1054,2	1382,4	4435,8	4835,9	4517	2478,9	2550
ENSMUSG00000030745	Il21r	6965,1	8295,6	7639,2	10284,3	11923,9	10338,7	9108,5	10035,1
ENSMUSG00000026285	Pdcd1	2987,7	3235	2387,1	4125	4404,7	4028,4	3529	3227,2
ENSMUSG00000031132	Cd40lg	12478,8	12018,1	9179,3	10888	10074,3	9027,4	11530,8	10820,5
ENSMUSG00000029075	Tnfrsf4	1195,2	1399,1	1594,6	1770,9	1948,1	2433,3	1543,3	1788,2
ENSMUSG00000004040	Stat3	15641,6	16941,8	16746,4	16275,1	19972,8	17312,8	15800,7	17732,4
ENSMUSG00000027985	Lef1	11400	13332,1	10270,6	13652,3	16393,3	13194	11678,3	12735,2
ENSMUSG00000021756	Il6st	1045,9	1202,5	1724,1	5375,5	5501,1	6317,7	2789,2	2721,5
ENSMUSG00000022661	Cd200	317,6	252,9	209,3	1210,2	1067,8	1245,4	593,5	646,1
ENSMUSG00000021356	Irf4	364,2	389,7	457,6	610	586,9	637,5	580,5	470,4
