# Supplementary material for: Stable characteristics of intrapopulation heterogeneity in virus-specific Th1 cells during chronic viral challenge infection
Source: Front Immunol. 2025 Dec 19;16:1716422. doi: 10.3389/fimmu.2025.1716422 (PMC12757370; doi:10.3389/fimmu.2025.1716422)
Supplement: Supplementary file 2 [file DataSheet1.pdf]

Supplementary Material

1 Supplementary Figures

1.1 Supplementary Figure 1: Phenotypic features of antiviral T-bet^{+} primary effector T cells

1.2 Supplementary Figure 2: Th1 characteristics of the progeny of antiviral T-bet reporter-sorted CD4^{+} T cells after LCMV Clone 13 challenge

1.3 Supplementary Figure 3: Phenotypic markers of exhaustion assessed in the progeny of antiviral T-bet reporter-sorted CD4^{+} T cells after LCMV Clone 13 challenge

2 Supplementary Methods

2.1 Antibody list

1 Supplementary Figures

1.1 Supplementary Figure 1: Phenotypic features of antiviral T-bet^{+} primary effector T cells

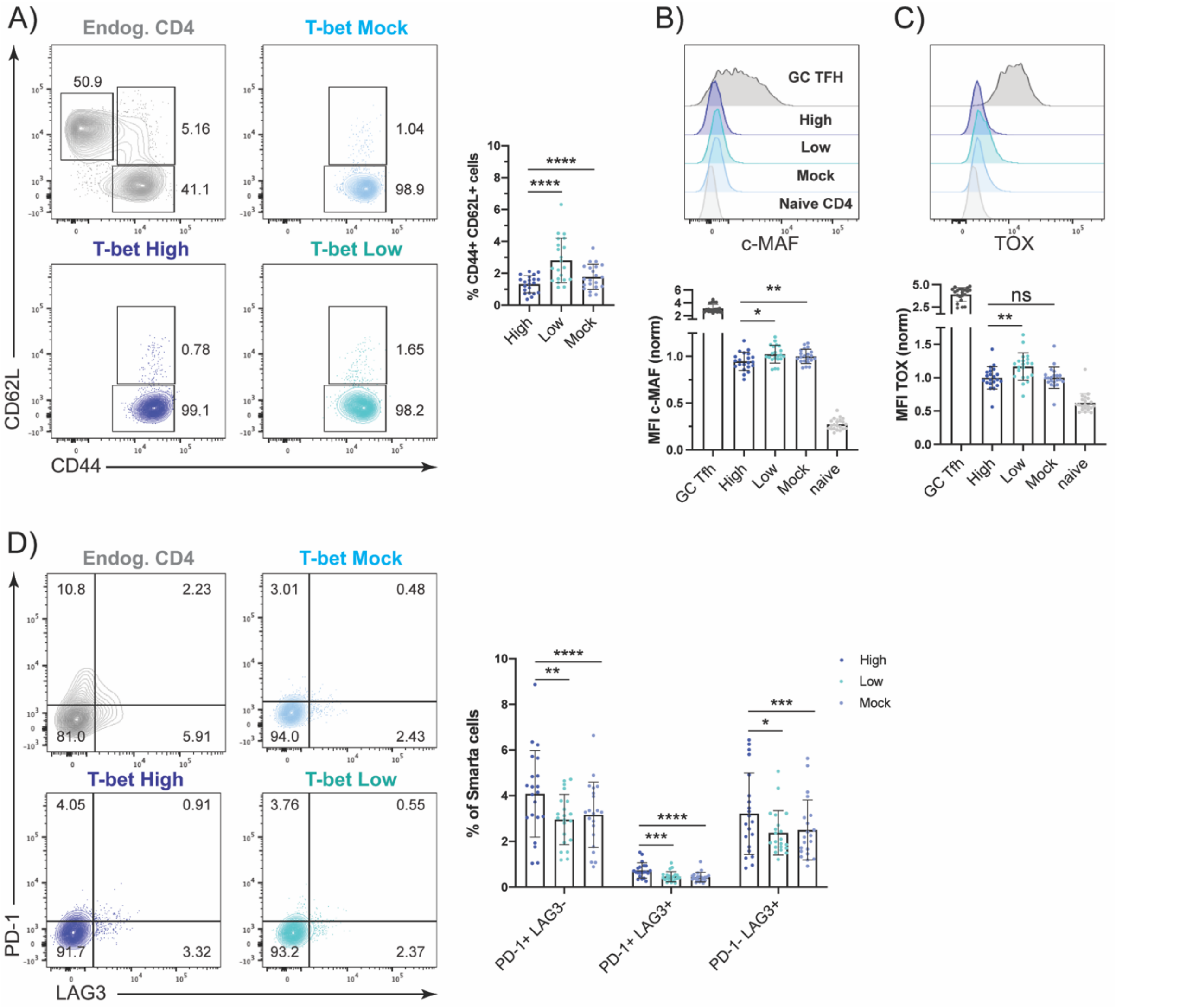

Supplementary Figure 1. Naïve Smarta CD4^{+} T cells from T-bet ZsGreen donors (Thy1.1^{+}) were transferred into T-bet ZsGreen recipients (Thy1.2^{+}). Recipient mice were infected with LCMV Arm (200pfu). On day 10 p.i. the cells were harvested from the spleen and lymph nodes. T-bet ZsGreen^{+} Smarta cells were electronically gated (egated) according to their T-bet reporter expression levels into T-bet^{high} or T-bet^{low} fractions, and all T-bet reporter positive cells (T-bet^{mock}) served as controls. (A) Representative gating of CD44 and CD62L of endogenous CD4^{+} T cells (grey) or the T-bet reporter sorted Smarta cells (shades of blue). Pooled CD44^{+}CD62L^{+} frequencies of Smarta cells. (B) Representative histogram of c-MAF expression (grey = naïve endog. CD4^{+} T cells, dark grey = endogenous effector GC Tfh (PD-1^{+}CXCR5^{+}) cells). Pooled and normalized c-MAF MFI of each egated fraction. (C) Representative histogram of TOX (grey = naïve endog. CD4^{+} T cells, dark grey = endogenous effector GC Tfh (PD-1^{+}CXCR5^{+}) cells). Pooled and normalized TOX MFI of each egated fraction. (D) Representative gating of PD-1 and LAG3 of endogenous CD4^{+} T cells (grey) or the egated fractions of Smarta cells (shades of blue). Pooled frequencies of different subsets. Data are

presented as mean ± SD. Each dot represents isolated Smarta T cells or endogenous CD4 T cells (grey) from one individual recipient. 5 independent experiments were pooled (n=4-5 mice/experiment). For MFI comparison, MFI of T-bet^{High}, T-bet^{Low}, endogenous GC Tfh or naïve CD4 T cells were normalized to the corresponding T-bet^{Mock} sample. Statistical significance was determined by paired T-test or Wilcoxon test comparing T-bet low or mock to high Smarta cell fraction, statistical comparison to endogenous cells was not performed. p* <0.05, p**< 0.01, p***< 0.001, p****< 0.0001, ns = not significant.

1.2 Supplementary Figure 2: Th1 characteristics of the progeny of antiviral T-bet reporter-sorted CD4⁺ T cells after LCMV Clone 13 challenge

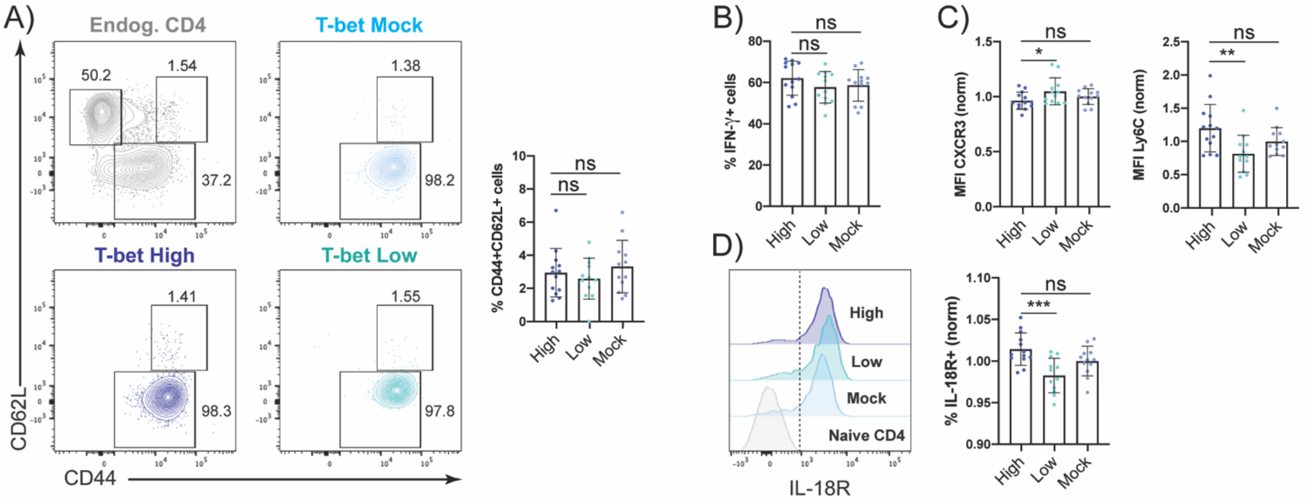

Supplementary Figure 2. Ten days p.i. with LCMV Arm, T-bet^{High}, T-bet^{Low} and T-bet^{Mock} sorted Smarta cells (Thy1.1^{+}) were transferred into individual naïve T-bet ZsGreen recipients (Thy1.2^{+}). Two weeks post transfer, the recipients were infected with high dose LCMV Clone 13 (≥2x10^{6} pfu) and 7 days post infection, the transferred cells were isolated from spleen and their phenotype was analyzed with flow cytometry. (A) Representative gating of CD44 and CD62L of endogenous CD4^{+} T cells (grey) or Smarta cells (shades of blue). Pooled frequency of CD44^{+}CD62L^{+} Smarta cells. (B) Pooled frequencies of IFN-γ^{+} Smarta CD4^{+} T cells after GP64-restimulation *ex vivo*. (C) Normalized and pooled MFI of CXCR3 and Ly6C of Smarta CD4^{+} T cells. (D) Representative histogram of IL-18R expression of Smarta CD4^{+} T cells (shades of blue) or naïve endogenous CD4^{+} T cells (grey). Normalized and pooled frequencies of IL-18R^{+} Smarta CD4^{+} T cells. Data are presented as mean ± SD. Each dot represents isolated Smarta T cells from one individual recipient. 3 independent experiments were pooled (n=4-5 mice/fraction/experiment). For MFI or IL-18R^{+} % comparison, MFI or frequencies of T-bet^{High} or T-bet^{Low} cells were normalized to the average of T-bet^{Mock} samples in each experiment. Statistical significance was determined by unpaired T-test or Mann-Whitney test comparing T-bet low or mock to high fraction. $p^{*} < 0.05$, $p^{**} < 0.01$, $p^{***} < 0.001$, ns = not significant.

1.3 Supplementary Figure 3: Phenotypic markers of exhaustion assessed in the progeny of antiviral T-bet reporter-sorted CD4^{+} T cells after LCMV Clone 13 challenge

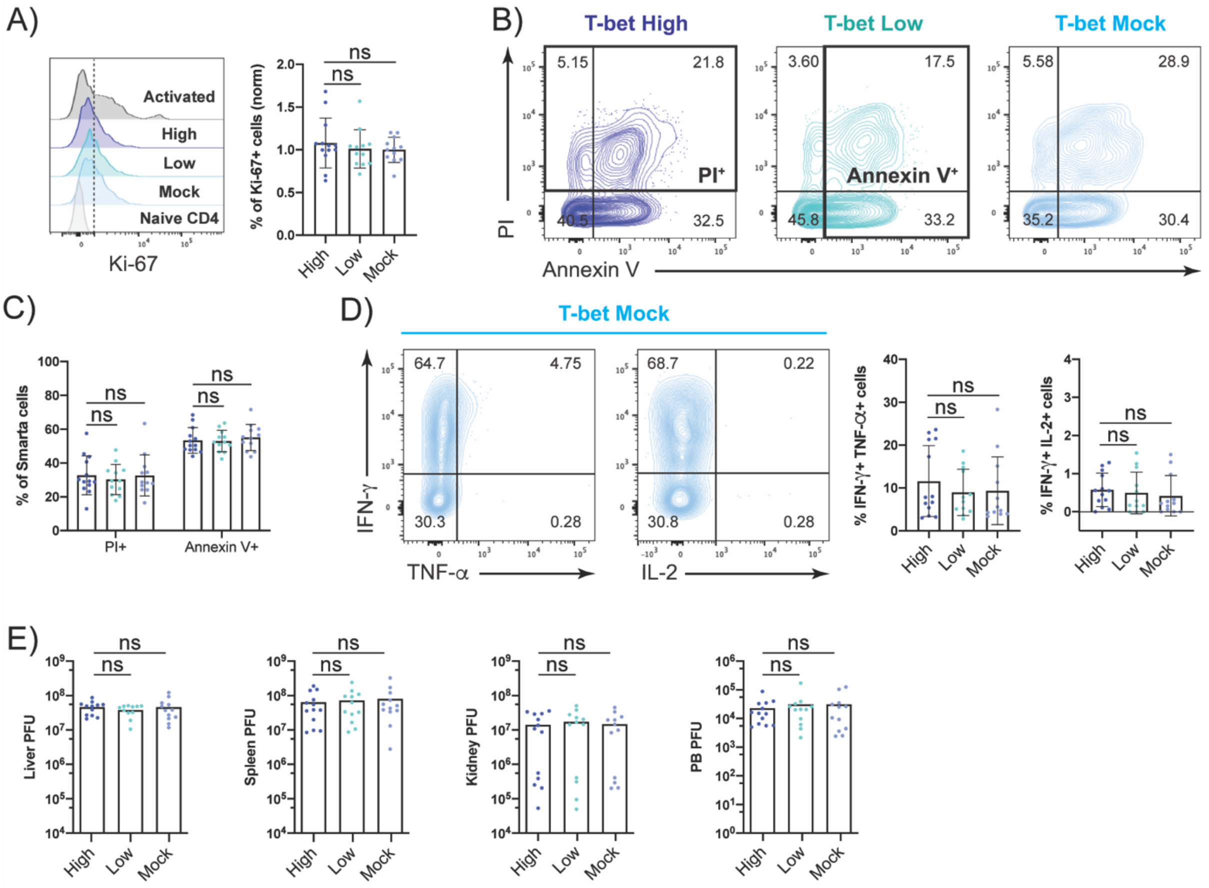

Supplementary Figure 3. Ten days p.i. with LCMV Arm, T-bet^{High}, T-bet^{Low} and T-bet^{Mock} sorted Smarta cells (Thy1.1^{+}) were transferred into individual naïve T-bet ZsGreen recipients (Thy1.2^{+}). Two weeks post transfer, the recipients were infected with high dose LCMV Clone 13 (≥2x10^{6} pfu) and 7 days post infection, the transferred cells were isolated from spleen and their phenotype was analyzed with flow cytometry. (A) Representative histogram of Ki-67 expression of Smarta CD4^{+} T cells (shades of blue) or endogenous naïve (light grey) or activated (dark grey) CD4^{+} T cells. Normalized and pooled frequencies of Ki-67^{+} Smarta CD4^{+} T cells. (B) Representative gating of Annexin V and PI of Smarta CD4^{+} T cells. (C) Pooled frequencies of dead (PI^{+}) or apoptotic (AnnexinV^{+}) Smarta cells. (D) Representative gating of IFN-γ and TNF-α or IL-2 coexpression of T-bet^{Mock} Smarta CD4^{+} T cells after GP64-restimulation *ex vivo*. Pooled frequencies of IFN-γ^{+}TNF-α^{+} and IFN-γ^{+}IL-2^{+} Smarta cells. (E) Pooled viral titers (plaque forming units, PFU) of liver, spleen, kidney and peripheral blood (PB). Data are presented as mean ± SD. Each dot represents isolated Smarta CD4^{+} T cells from one individual recipient. 3 independent experiments were pooled (n=4-5 mice/fraction/experiment). For comparison of Ki-67^{+} cell frequencies, frequencies of T-bet^{High} or T-bet^{Low} cells were normalized to the average of T-bet^{Mock} samples in each experiment. Statistical significance was determined by unpaired T-test or Mann-Whitney test comparing T-bet low or mock to high fraction. ns = not significant.

2 Supplementary Methods

2.1 Antibody list

Target	Conjugate	Clone	Company	Cat. Number	RRID
Bcl6	A647	K112-91	BD	561525	AB_10898007
IL-18Ra	A647	A17071D	BioLegend	157907	AB_2860736
Ki-67	A647	B56	BD	561126	AB_10611874
T-bet	AF488	4B10	Biolegend	644829	AB_2566018
Thy1.1	AF700	OX-7	Biolegend	202527	AB_1626244
Annexin V	APC	-	BD	550475	AB_2868885
LAG3	APC	C9B7W	eBioscience	17-2231-80	AB_2573183
PD-1	APC	J34	eBioscience	17-9985-80	AB_11149860
TNF	APC	MP6-XT22	BD	561062	AB_398553
Thy1.2	APC-Cy5	Ho13	In House		
CD4	APC-Cy7	GK1.5	BD	565650	AB_2739324
CD44	APC-Cy7	IM7	BioLegend	103027	AB_830784
CXCR5	Biotin	L138D7	BioLegend	145509	AB_2562125
CD8	Biotin	53-6.7	In House		
CD11b	Biotin	M1/70	BD	553309	AB_394773
CD11c	Biotin	HL3	BD	553800	AB_395059
CD25	Biotin	7D4	In House		
Gr-1	Biotin	RB6-8C5	BD	553125	AB_394641
CD19	Biotin	1D3	In House		
CXCR3	Biotin	CXCR3-173	eBioscience	13-1831-82	AB_1210592
NK1.1	Biotin	PK136	BD	553163	AB_394675
T-bet	BV421	4B10	Biolegend	644832	AB_2686976
Tcf1/Tcf7	BV421	S22-966	BD	566692	AB_2869822
CD62L	BV605	Mel-14	Biolegend	104437	AB_11125577
CD4	BV650	RM4-5	BioLegend	100545	AB_11126142
PD-1	BV785	29F.1A12	Biolegend	135225	AB_2563680
IFNg	eFluor450	XMG1.2	eBioscience	48-7311-80	AB_1834367
CXCR3	PB	CXCR3-173	Biolegend	126529	AB_2563100
Thy1.1	PB	OX-7	In House		
CD4	PE	GK1.5	BD	553730	AB_396634
LAG3	PE	C9B7W	Biolegend	125207	AB_2133344
Tox	PE	TXRX10	eBioscience	12-6502-82	AB_10855034
PD-1	PE	J43	eBioscience	12-9985-81	AB_466294

CD4	PE-Cy7	RM4-5	BD	552775	AB_394461
CD62L	PE-Cy7	MEL-14	eBioscience	25-0621-82	AB_469633
IL2	PE-Cy7	JES6-5H4	eBioscience	25-7021-82	AB_1235004
Ly6C	PE-Cy7	HK1.4	eBioscience	25-5932-82	AB_2573503
CXCR5	PE-Vio770	REA215	Miltenyi	130-117-366	AB_2733206
c-Maf	PerCP	sym0F1	eBioscience	46-9855-42	AB_2573908
CXCR3	PerCP	CXCR3-173	eBioscience	45-1831-82	AB_1210699
Ly6C	PerCP	HK1.4	eBioscience	45-5932-82	AB_2723343
Streptavidin	PerCP	-	BD	554064	AB_2336918
Thy1.1 (OX-7)	PerCP	OX-7	BD	557266	AB_396611
CD8	V500	53-6.7	BD	560776	AB_1937317
